# Serological evidence of MERS-CoV and HKU8-related CoV co-infection in Kenyan camels

**DOI:** 10.1080/22221751.2019.1679610

**Published:** 2019-10-24

**Authors:** Wei Zhang, Xiao-Shuang Zheng, Bernard Agwanda, Sheila Ommeh, Kai Zhao, Jacqueline Lichoti, Ning Wang, Jing Chen, Bei Li, Xing-Lou Yang, Shailendra Mani, Kisa-Juma Ngeiywa, Yan Zhu, Ben Hu, Samson Omondi Onyuok, Bing Yan, Danielle E. Anderson, Lin-Fa Wang, Peng Zhou, Zheng-Li Shi

**Affiliations:** a CAS Key Laboratory of Special Pathogens, Wuhan Institute of Virology, Center for Biosafety Mega-Science, Chinese Academy of Sciences, Wuhan, People's Republic of China; b University of Chinese Academy of Sciences, Beijing, People’s Republic of China; c Department of Zoology, National Museums of Kenya, Nairobi, Kenya; d Institute for Biotechnology Research, Jomo Kenyatta University of Agriculture and Technology, Nairobi, Kenya; e Directorate of Veterinary Services, State Department of Livestock, Ministry of Agriculture, Livestock and Fisheries, Kenya; f Kenya Camel Association, Nairobi, Kenya; g Programme in Emerging Infectious Diseases Duke-NUS Medical School, Singapore, Singapore

**Keywords:** MERS, coronavirus, bat, camel, HKU8

## Abstract

Dromedary camels are important reservoir hosts of various coronaviruses, including Middle East respiratory syndrome coronavirus (MERS-CoV) that cause human infections. CoV genomes regularly undergo recombination during infection as observed in bat SARS-related CoVs. Here we report for the first time that only a small proportion of MERS-CoV receptor-binding domain positive (RBD) of spike protein positive camel sera in Kenya were also seropositive to MERS-CoV nucleocapsid (NP). In contrast, many of them contain antibodies against bat HKU8-related (HKU8r)-CoVs. Among 584 camel samples that were positive against MERS-CoV RBD, we found only 0.48 (8.22%) samples were also positive for NP. Furthermore, we found bat HKU8r-CoV NP antibody in 73 (12.5%) of the MERS-CoV RBD positive and NP negative samples, yet found only 3 (0.43%) of the HKU8r-CoV S1 antibody in the same samples. These findings may indicate co-infection with MERS-CoV and a HKU8r-CoV in camels. It may also raise the possibility of the circulation of a recombinant coronavirus virus with the spike of MERS-CoV and the NP of a HKU8r-CoV in Kenya. We failed to find molecular evidence of an HKU8r-CoV or a putative recombinant virus. Our findings should alert other investigators to look for molecular evidence of HKU8r-CoV or recombinants.

## Introduction

Since September 2012, Middle East Respiratory Syndrome (MERS) has spread from the Middle East to 27 countries, causing more than 2374 laboratory-confirmed cases of infection with an average mortality of 35.4% (as of February 2019) [1] . The WHO recommendation for laboratory detection of the causal agent, MERS coronavirus (MERS-CoV) includes real-time RT–PCR targeting upstream of the E protein gene or nucleocapsid protein (NP) gene, complemented with ORF1a and ORF1b [2]. Serological tests are also recommended when a viral nucleic acid test was not applicable, or for population-based serosurveys and investigations of past exposures. Viral spike (S) protein-based indirect ELISA followed by a confirmatory virus neutralization test (VNT) is the mostly widely used serological test for MERS-CoV. In contrast, NP based serological assays are seldom used because of their greater cross-reactivity [2–7].

Tracing the source of human MERS infection is pivotal to prevent transmission of this disease. Dromedary camels have been identified as an important source for zoonotic transmission of MERS-CoV after detection of neutralizing antibodies (76–100% seropositive) and almost identical viruses were identified in Middle East camels [3,8]. It is widely believed that MERS-CoV could have originated from Africa as most of these animals were originally bred and exported from Africa [9]. Although, MERS-CoV neutralizing antibodies and viral RNA have been found in camels from West and East African countries, zoonotic human infections have not been reported in Africa [4,9,10,11]. The majority of these studies used an S-based serosurvey and showed high positivity for MERS-CoV infection in camels [3,4,8,9,11].

There are two studies which have previously tested both S and NP IgG antibodies in Middle East camels. Of the two studies, one reported a strong correlation between NP positives by Western blot (97%) and S-based VNT positives (98%) using camel samples from the United Arab Emirates [12]. The second study tested camel sera from Saudi Arabia, and found more samples seropositive for S than NP by ELISA, Western blot or the Luciferase Immunoprecipitation System (LIPS) [13]. The latter study did not proceed with further analysis.

In this study, we conducted a nationwide serosurvey for coronavirus infection from samples first screened for MERS-CoV by ELISA and VNT [14]. Our aim was to investigate the possible new genotype of MERS-CoV in Kenyan camels using a serology-predicted pathogen discovery strategy.

## Materials and methods

### Sample collection

The Director of Veterinary Service at the Ministry of Agriculture Livestock and Fisheries in Kenya granted permission to conduct this study. The camel sampling was performed in 2016 and 2018 across 13 counties where camels are reared, taking into account breed types [14]. Four distinct breeds of camel are recognized in Kenya: Somali, Turkana, Gabbra/Rendille, and Imported Pakistani (which were imported from Pakistan in the 1980s). The sampling locations were recorded and confirmed by GPS. Other information such as age, sex, pregnancy, and migration was collected. A total of 894 camel serum samples were used in this study. In addition, 1136 camel nasal swabs collected in January–February 2016 and 286 camel nasal swabs collected in February–March 2018 were used for molecular tests. All camel samples were collected in compliance with local regulations, and then transported to Wuhan Institute of Virology (WIV) according to IATA international regulations for transporting biological samples [14].

### Serological tests

An in-house anti-MERS-CoV IgG ELISA kit was developed based on the purified receptor-binding domain (RBD) of spike protein, and validated for Kenya and Pakistan camels [14,15]. Purified NP of MERS-CoV, SARS-like CoV Rp3, human coronaviruses (OC43, HKU1, 229E, and NL63), and bat CoVs (HKU2 and HKU9) were produced and applied to ELISA or Western blot in this and previous studies [16–18]. A bat HKU8r-CoV (ID: 4050 from China) NP gene that shares a 93% amino acid sequence identity to the *Miniopterus* bat coronavirus HKU8-CoV NP (GenBank accession number YP_001718616), was inserted into pET-28a+ (Novagen) for prokaryotic expression. Kenyan HKU8r-CoV strain BtKy33 NP and S1 (synthesized from GenBank accession no.HQ728485.1) were inserted into pCAGGS or pHCMV vector with N-terminal S-tag. BtKy33 NP and S1 plasmids transiently transfected HEK293T-17 cell supernatant was used in Western blot. Camel serum samples were tested in the ELISA (1:20 dilution) or Western blot (1:100 dilution) and goat anti-camel IgG-HRP conjugate (Alpha Diagnostic International) was used as the secondary antibody at 1:3000 dilution. A cut-off value for each antigen was determined in ELISA after validation.

Lysates of MERS-CoV infected Vero cells were generated in the biosafety level 3 laboratory at WIV, loaded onto 12% SDS-PAGE gels, and transferred onto nitrocellulose membranes. Membranes were incubated with selected MERS-CoV RBD positive and NP positive or negative camel sera for 1 h at 37°C (1:100 dilution) after blocking. Membranes were then washed and then incubated with anti-camel IgG-HRP secondary antibody (as above) for another 1 h at 37°C, followed by three more washes.

MERS-CoV NP, China HKU8r-CoV NP (above) and S1 (amino acid 1–150 of S protein) (synthesized from GenBank accession no. YP_001718612.1) were codon-optimized and inserted in the pREN2 vector [15,19]. Plasmids were transfected into HEK293T-17 cells using Lipofectamine 3000 (Thermo Fisher Scientific). Cells were then collected, lysed and incubated with camel serum samples. Serum (1 μl) was incubated with 10 million units of Rluc alone (vector) or Rluc-N or S1, respectively, together with 3.5 μl of a 30% protein A/G UltraLink resin suspension (Pierce, Thermo Fisher Scientific). The ratio of Rluc-N or S1: Rluc (vector) was used to determine the specific antigen reactivity of camel sera. HKU8r-CoV S1 protein was expressed from the pCAGGS vector and was purified using S-tag resin (generated in-house). Mouse anti-serum against purified protein was used as a positive control in LIPS.

### Molecular detection

Viral RNA was extracted from camel nasal swabs using a viral RNA extraction kit (Roche, Germany) according to the manufacturer's instructions. Three primer pairs were used to screen the samples in RT–PCR, two targeting the conserved RNA-dependent RNA polymerase gene of CoVs and another targeting the MERS-CoV S2 region [20,21]. Twelve pools of RNA were made from 139 MERS-CoV negative samples (roughly every 10 samples were pooled) and libraries for next-generation sequencing were prepared using Illumina Truseq mRNA kit (TruSeq Stranded mRNA Library Prep Kit, Cat # RS-122-2101) following the manufacturer's instructions. The sequencing was performed on a HiSeq 3000 sequencer and data was analysed using the Galaxy platform.

### Statistical analysis

All analyses were performed using IBM SPSS Statistics (version 25). Two-tailed Mann–Whitney *U* exact test and two-tailed Student's *t* exact test were used to calculate the 95% confidence interval (CI) of positive rate. The association *p* values between viral seropositive samples and camel information were calculated using Chi-square test followed with Yates correction two-tailed test and Fisher's exact test.

## Results

**Figure 1:**
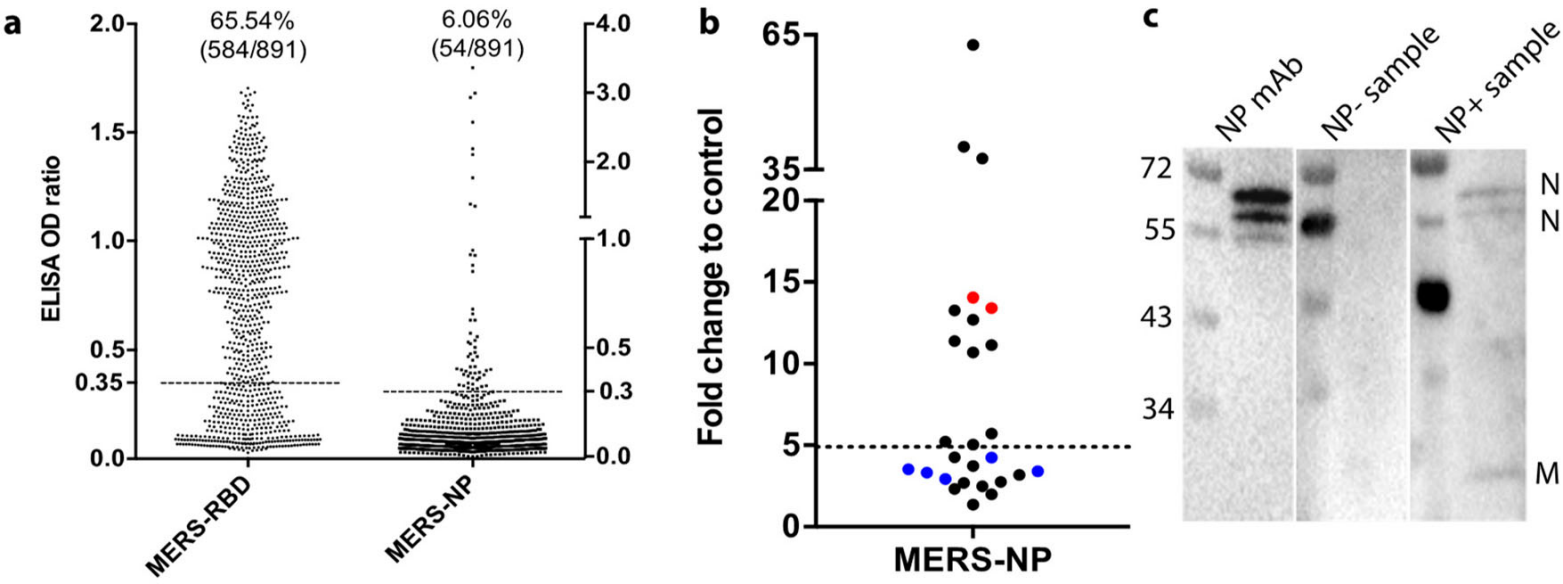
. Absence of MERS-CoV nucleotide protein (NP) antibody in most of the MERS-CoV receptor-binding domain (RBD) positive camel sera. (A) ELISA screening of MERS-CoV RBD and NP antibodies (*n* = 891 camels). Cutoff (dashed line, same as below) shown as five times the mean value of the negative samples, it was further adjusted to 0.35 for RBD in line with results from neutralization assay. (B) Luciferase Immunoprecipitation System (LIPS) test for NP antibodies. Eight NP ELISA positive and 12 negative samples (all MERS-CoV RBD positive) were used in the LIPS test. Five RBD and NP ELISA negative samples were used as controls (shown as blue dots), and alpaca anti-serum against MERS-CoV was used as positive controls (red dots). Cutoff was determined as the sum of mean value plus three times the standard deviations of the negative samples. (C) Western blot using whole virus proteins. Lysates of MERS-CoV infected Vero cells were separated on 12% polyacrylamide gel, transferred to nitrocellulose membranes and analysed with MERS-CoV RBD positive, NP positive/negative camel serum samples, or NP mAb. MP, membrane protein. Molecular markers were indicated (kDa). Information for sample applied to WB, LIPS or ELISA can be found in Supplementary Table 1.

We previously performed a nationwide serosurvey for MERS-CoV in Kenyan camels using an in-house MERS-CoV RBD IgG ELISA, plus a confirmatory VNT [14]. A correlation in results obtained using the two methods was observed whereby almost all ELISA positive sera were capable of neutralizing MERS-CoV (103/105, 98.1%). Our data revealed 584 of 891 (65.54%) Kenyan camel samples had MERS-CoV RBD antibodies (Figure 1(A)). We then tested all camel sera using a MERS-CoV NP-based ELISA. In contrast, only 54 of 891 samples (6.04%), or 48 of the RBD positive samples (8.22%) were NP positive (Figure 1(A)). To confirm the finding, we developed an additional antibody assay based on mammalian expressed MERS-CoV NP using LIPS, a fast and sensitive serological tool which has previously been successfully used in SADS-CoV serosurvey [18,19]. All NP ELISA positive sera were also positive in LIPS, while those negative samples remained negative (Figure 1(B)). This observation was confirmed by a MERS-CoV virus-based Western blot using RBD positive samples. Two major structural proteins, NP and membrane protein were observed in the NP positive group, but not in the NP negative group (Figure 1(C) and supplementary Figure 1(A)). Above all, only a small proportion of the MERS-CoV RBD positive serum samples were also NP positive, different to that observed in human infection cases [6,22]. This observation indicated that the majority of the camels may only maintain NP antibodies for a short time or be co-infected by another CoV.

**Figure 2:**
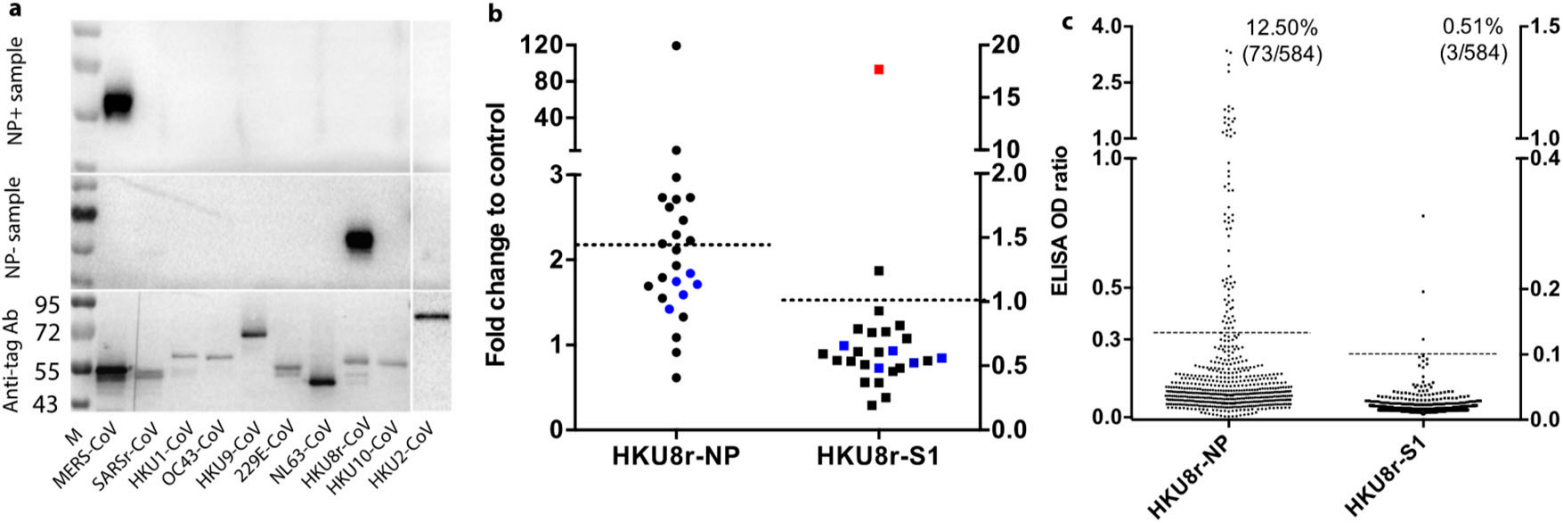
. Presence of HKU8r-CoV antibodies in MERS-CoV RBD positive camel sera. (A) Coronavirus NP protein array. Successful expression was determined using anti-HIS tag (all CoVs except HKU2-CoV) or anti-FLAG tag (HKU2-CoV) antibodies. Samples seropositive (C0184) or negative (C0039) for MERS-CoV NP were used. Markers were indicated (kDa). (B) LIPS test for HKU8r-CoV (China strain) NP and S1 antibodies. Twelve NP ELISA positive and eight negative samples (all MERS-CoV RBD positive) were used in LIPS test. Five MERS-CoV RBD and NP ELISA negative samples were used as control in each panel (shown as blue dots), and rabbit anti-serum against HKU8r-CoV S1 protein was used as positive control (red dot). Cutoff was determined as the sum of mean value plus three times the standard deviations of the negative samples. (C) ELISA screening for HKU8r-CoV (China strain) NP and S1 antibody in MERS-CoV RBD positive camel serum samples (*n *= 584). Cutoff shown as five times the mean value of the negative samples. Information for sample applied to WB, LIPS or ELISA can be found in Supplementary Table 1.

In searching for possible new genotype of MERS viruses that share the same or a related S but a different NP, we performed a CoV NP protein array serology test. Selected MERS-CoV RBD positive samples were analysed by Western blot using NP proteins from representative alpha and beta CoVs (see method section). The successful expression of these proteins was confirmed by anti-tag antibodies (Figure 2(A)). No other CoV besides HKU8r-CoV (China strain) NP was detected in two out of three selected MERS-CoV NP negative camel serum samples (Figure 2(A) and Supplementary Figure 1(B)). In particular, HKU8r-CoV NP antibody was not cross-reactive with 229E-related CoV, which is also known to be enzootic in camels (Figure 2(A)). HKU8r-CoV has previously been found only in *Miniopterus* bats in China or Kenya [23–25].

**Figure 3:**
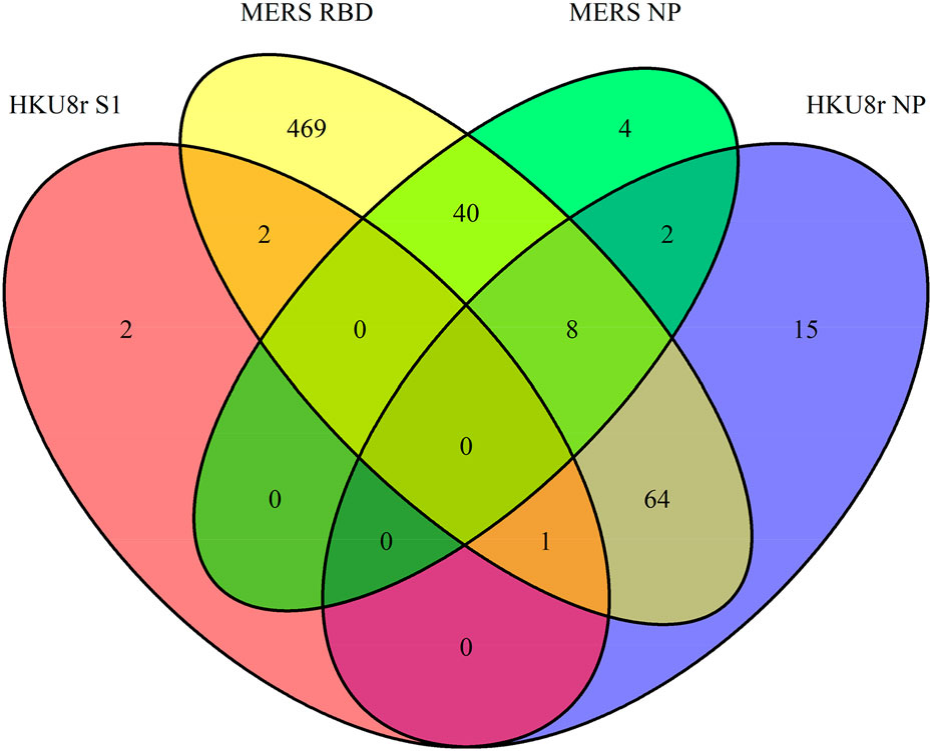
. Venn diagram for virus serum positivity of MERS-CoV or HKU8r-CoV. Totally 605 out of 891 camel sera that were positive for at least one of the viral proteins were included.

To further investigate the results observed in the Western blot, HKU8r-CoV NP and S1 based LIPS assays were developed. The LIPS results demonstrated clear HKU8r-CoV NP positive in some camel sera, consistent with Western blot results (Figure 2(B)). However, no HKU8r-CoV S1 positives were found within the HKU8r-CoV NP positive samples (Figure 2(B)). To rule out the possible false-negative results using China HKU8r-CoV as detection antigen, a Kenyan bat HKU8r-CoV BtKY33 was also used. Results indicated that the above conclusion was not HKU8r-CoV strain-specific (Supplementary Figure 2). The data indicates that some of the Kenyan camels have both MERS-CoV S and HKU8r-CoV NP antibodies. We then performed a nationwide serosurvey for HKU8r-CoV in camels using a HKU8r-CoV NP and S1 ELISA. Of the 584 MERS-CoV RBD positive samples tested, 73 (12.5%) were positive for HKU8r-CoV NP (Figure 2(C)). In contrast, only 3 (0.51%) were seropositive for HKU8r-CoV S1 (Figure 2(C)). The data suggested the idea of the co-infection of MERS/HKU8r-CoVs, alternatively, we can't rule out the existence of a chimeric MERS-CoV carrying MERS-CoV S gene but a NP gene from HKU8r-CoV in Kenyan camels (Figure 3 and Supplementary Table 1). Although, we cannot rule the possibility of a novel CoV that was not detected in molecular assays but can cross-react with HKU8r or MERS coronavirus positive serum samples. It is worth to mention, another camel alphacoronaviruses, 229E-like coronaviruses were previously found in camels [26]. We then tested cross-reactivity of antibodies to the N proteins of HKU8r and 229E as the two are closely related phylogenetically. Our data demonstrated only 10/90 of the HKU8r-CoV NP positive camel serum were also 229E-NP positive (Supplementary Figure 3(A)), which was probably true positive for the later as NP of the two viruses showed no cross-reactivity using positive control samples (Supplementary Figure 3(B)).

We then investigated factors that may associate with MERS-CoV (positive for both S and NP) seropositivity and distribution, which may also be important for the detection of this MERS/HKU8r-CoV co-infection cases. Distribution of MERS-CoV sero-positive samples was not found to be associated with sex, age, birthplace, migration or pregnancy of camels but may be associated with the breed (*P *= .003) (Supplementary Table 2).

We also screened nucleic acid by RT–PCR targeting the conserved regions of all known coronaviruses on 1422 camel nasal swabs collected from Kenya 2016–2018. In addition, 12 pooled camel nasal swabs (10 samples for each pool) RNA were also tested in next-generation sequencing (NGS). We found MERS-CoV nucleic acid by both RT–PCR and NGS, yet so far we have not found nucleic acid positive for HKU8r-coronavirus.

## Discussion

In a nationwide MERS-CoV sero-surveillance of Kenyan camels, we have demonstrated that most of the MERS-CoV RBD positive samples (91.78%) were not MERS-CoV NP positive by multiple serological assays. In contrast, 12.5% of the MERS-CoV RBD positive samples also carried NP antibodies to HKU8r-CoV, a virus that has only so far been found in bats. For the first time, we demonstrate the presence of a HKU8r-CoV infection in camels.

Our study is the first to compare the seroprevalence of MERS-CoV in camels using both S and NP serological assays. As one of the four major structural proteins in CoV, NP is widely used as a diagnostic target due to its high abundance upon infection. Although NP showed good reactivity with human MERS patient serum in some studies, S-based serological tests are recommended by WHO for diagnosis due to its greater specificity [2]. In line with these recommendations, most camel serosurveys were conducted using only S-based assays [3,8,9,11,13]. In human cases, samples showing neutralizing activity were also NP positive [6,22]. However, our data clearly demonstrate that most of the MERS-CoV neutralizing samples were not MERS-CoV NP positive in camels. Similarly, unmatched S and NP positive serological patterns have also been observed in another study using Saudi Arabia camel serum samples [13]. A possible interpretation is that the duration of the NP antibody responses is more short-lived than S antibody in camels.

The seropositive of HKU8r-CoV in camels is another example linking a bat CoV to camels besides MERS-CoV and 229E-related coronavirus, thereby strengthening the bat-camel connection theory [27,28]. The finding of two closely related NeoCoV and PREDICT/PDF-2180 CoV in bats suggested that MERS-CoV descended from an ancestral virus of African bats, while the host switch from bats to dromedary camels likely occurred in Africa years ago [27,29,30]. However, there has been no other reported virus spillover from bats to camels beside this ancestral MERS-CoV and 229E-related coronaviruses. Our data demonstrated that the HKU8r-CoV NP antibody is not caused by cross-reactive antibody responses to camel 229E. Our discovery of HKU8r-CoV in camels is, therefore, an important finding. HKU8r-CoV was described first in Hong Kong and later in Kenya, and so far it has only been reported in *Miniopterus* bat species [23–25]. Kenyan bats carry multiple CoVs including SARS-related, HKU2-related, HKU7-related, HKU8-related and HKU9-related CoVs that may spillover to other species in rural areas where bats roost in close proximity to humans or are consumed by humans as bush meat [24].

It is worth mentioning that we observed much less HKU8r-CoV S protein IgG antibodies in any of the HKU8r-CoV NP positive and MERS-CoV RBD positive samples. The chance of MERS/HKU8r-CoV co-infected camels simultaneously lost both HKU8r-CoV S and MERS-CoV NP antibodies should be low. The presence of a chimeric MERS/HKU8r-CoV would be a possible explanation, although there could be other explanations. For example, camel may generate a different S and NP antibody responses that resulted in a stronger S but weak and short-lasting NP antibody titres. It could also be two separated events of infection by MERS-CoV and HKU8r-CoV instead of a co-infection. Nonetheless, even the occurrence of a chimeric MERS/HKU8r-CoV is not surprising as it is known that CoV genomes regularly undergo recombination during infection as observed in CoVs such as bat SARS-related CoVs [31]. Three CoVs, including MERS-CoV, 229E-like and HKU23-CoV had also been reported to co-circulate in Middle East camels, increased chance of recombination [28]. Yet it is worth noting that recombination between an alpha-CoV (HKU8r-CoV e.g.) and beta-CoV (MERS-CoV e.g.) has never been reported. Our observation should be more supported by molecular evidence in future studies.

Molecular detection of these serology-predicted novel CoVs containing MERS-CoV S protein is hampered by the lack of genetic sequences of this possible recombinant virus at the present time. Our Western blot results suggest at least some of the MERS-CoV neutralizing samples did not have NP or M protein antibodies. It is unknown what genetic backbone these viruses will have. For example, viruses may maintain the MERS-CoV backbone and only replace the NP gene with HKU8r-CoV (or unidentified CoVs) or vice versa. In the latter example, these viruses would have a HKU8r-CoV backbone with S gene replaced by the MERS-CoV. With this hypothesis in consideration, the current molecular diagnosis tools for MERS-CoV do not detect other genotype of MERS-CoVs circulating in animals, partially explaining the failure in our attempt to find molecular evidence of novel chimeric CoVs using qPCR targeting MERS-CoV S gene or metagenome NGS. Concerted efforts to identify and sequence these viruses are needed in future studies.

In conclusion, we showed evidence for HKU8r-CoV virus infection in camels in Kenya. It is possible that this virus co-circulates with MERS-CoV. But the possibility of a MERS-CoV-HKU8r recombinant virus cannot be excluded. Future research should seek to demonstrate molecular evidence of such viruses.

## Supplementary Material

Supplemental Material
