# Supplementary material for: Serological evidence of MERS-CoV and HKU8-related CoV co-infection in Kenyan camels
Source: Emerg Microbes Infect. 2019 Oct 24;8(1):1528–34. doi: 10.1080/22221751.2019.1679610 (PMC6818114; doi:10.1080/22221751.2019.1679610)
Supplement: Supplemental Material [file TEMI_A_1679610_SM2505.docx]

Supplementary Figure 1. Western blot using whole virus proteins (A) or coronavirus NP protein (B). Another camel sample was used to support the results shown in Figure 1C (camel sample number C0039) or Figure 2A (camel sample number C0047). Same settings were used. Molecular markers were indicated (kDa). Information for sample applied to WB can be found in Supplementary Table 1.


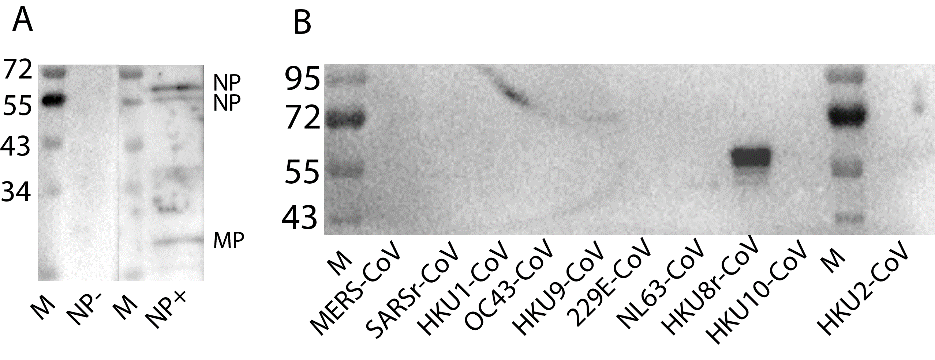


Supplementary Figure 2. Western blot using Kenyan HKU8r-CoV BtKy33 strain NP and S1 proteins. Nine MERS-CoV NP ELISA positive and eight negative samples (all MERS-CoV RBD positive) were used. Anti-S-tag antibody was used as positive control. Molecular markers were indicated (kDa). Information for sample applied to WB can be found in Supplementary Table 1.

Supplementary Figure 3. Cross-reactivity of HKU8r-CoV and 229E-CoV N proteins. (A) Ninety HKU8r-CoV NP ELISA positive were used in ELISA which used 229E-CoV N protein as detection antigen. Cut-off was set up as 0.2 according to five times the mean value of the negative samples. (B) Western blot figure shows cross-reactivity of the two NP using their respective positive serum samples. Anti-HIS-tag antibody was used as positive control. Two HKU8r-CoV positive camel serum samples (as HKU8r NP +1 and +2) and two human 229E-CoV positive serum samples (as 229E NP+1 and +2) were used.

Supplementary Table 1. Full list of ELISA screening of MERS-CoV RBD and NP, or

HKU8r-CoV S1 or NP. n=574 samples positive for MERS-CoV RBD. Strong positive (+++), OD value > 1; positive (+), OD value <1 but > cutoff; negative

(-), OD value < cutoff. ND=Not Done. *Samples that were also tested by WB;

#samples that were also tested by LIPS.

| Samples | MERS-CoV | | HKU8-CoV | |
| --- | --- | --- | --- | --- |
|  | RBD | NP | S1 | NP |
| No serum control | - | - | - | - |
| MERS-CoV infected Alpaca serum 1 | + | + | ND | ND |
| MERS-CoV infected Alpaca serum 2 | + | +++ | ND | ND |
| C0001 | + | - | - | + |
| C0002 | +++ | - | - | - |
| C0003 | + | - | - | - |
| C0004 | + | - | - | + |
| C0006 | +++ | - | - | - |
| C0007 | +++ | - | - | - |
| C0010 | +++ | - | - | - |
| C0011 | +++ | - | - | + |
| C0012 | +++ | - | - | - |
| C0013 | +++ | - | - | + |
| C0014 | +++ | - | - | - |
| C0017 | + | - | - | - |
| C0018 | + | - | - | - |
| C0019 | + | - | - | - |
| C0020 | + | - | - | - |
| C0023 | +++ | - | - | + |
| C0031 | +++ | - | - | +++ |
| C0032 | +++ | - | - | + |
| C0033 | +++ | - | - | - |
| C0035 | + | - | - | + |
| C0037 | + | - | - | + |
| C0039 | +++ | - | - | +++ |
| C0042 | +++ | - | - | - |
| C0043 | +++ | - | - | + |
| C0044 | +++ | - | - | - |
| C0045 | +++ | - | - | - |
| C0046 | +++ | - | - | - |
| C0047*^#^ | +++ | - | - | +++ |
| C0050 | +++ | - | - | - |
| C0052 | + | - | - | - |
| C0054 | + | - | - | - |
| C0055^#^ | +++ | - | - | +++ |
| C0057 | +++ | - | - | - |
| C0058 | +++ | - | - | - |
| C0059 | +++ | - | - | - |
| C0060 | +++ | - | - | - |
| C0061 | + | - | - | - |
| C0062 | +++ | - | - | - |
| C0063 | +++ | - | - | - |
| C0064 | +++ | - | - | - |
| C0066 | +++ | - | - | - |
| C0067 | +++ | - | - | + |
| C0068 | + | - | - | - |
| C0069 | +++ | - | - | - |
| C0070 | +++ | - | - | - |
| C0071 | +++ | - | - | - |
| C0073 | +++ | - | - | + |
| C0074 | +++ | - | - | - |
| C0075 | +++ | - | - | - |
| C0077 | + | - | - | - |
| C0078 | + | - | - | - |
| C0094 | + | - | - | - |
| C0098 | + | - | - | - |
| C0099 | +++ | - | - | - |
| C0100 | + | - | - | - |
| C0101 | +++ | - | - | - |
| C0104 | + | - | - | - |
| C0107 | + | - | - | - |
| C0110 | + | - | - | - |
| C0111 | +++ | - | - | - |
| C0112 | +++ | - | - | - |
| C0114 | + | - | - | - |
| C0123 | + | - | - | - |
| C0125 | + | - | - | - |
| C0126 | + | - | - | - |
| C0136 | + | - | - | - |
| C0140 | + | - | - | - |
| C0146 | + | - | - | - |
| C0147 | +++ | - | - | - |
| C0148 | + | - | - | - |
| C0149 | +++ | - | - | - |
| C0150 | +++ | - | - | - |
| C0151 | +++ | - | - | - |
| C0152 | +++ | - | - | - |
| C0153 | +++ | - | - | - |
| C0154 | +++ | - | - | - |
| C0155 | +++ | - | - | - |
| C0156 | +++ | - | - | - |
| C0157 | +++ | - | - | - |
| C0158 | +++ | - | - | - |
| C0159 | +++ | - | - | - |
| C0160 | +++ | - | - | - |
| C0161 | +++ | - | - | - |
| C0162 | +++ | - | - | - |
| C0163 | +++ | - | - | - |
| C0164 | +++ | - | - | - |
| C0166 | +++ | + | - | - |
| C0167^#^ | +++ | - | - | +++ |
| C0168 | +++ | - | - | - |
| C0169 | +++ | - | - | - |
| C0170 | +++ | - | - | + |
| C0171 | +++ | - | - | - |
| C0172^#^ | +++ | - | - | +++ |
| C0173 | +++ | - | - | - |
| C0174 | +++ | - | - | - |
| C0175 | +++ | - | - | - |
| C0176 | +++ | - | - | - |
| C0177 | +++ | - | - | - |
| C0179 | +++ | - | - | - |
| C0180 | +++ | - | - | - |
| C0182 | +++ | - | - | - |
| C0183 | +++ | - | - | - |
| C0184*^#^ | +++ | +++ | - | - |
| C0185 | +++ | - | - | - |
| C0186 | +++ | - | - | - |
| C0187^#^ | + | +++ | - | - |
| C0188 | +++ | - | - | - |
| C0189 | + | + | - | +++ |
| C0190 | +++ | - | - | - |
| C0191 | +++ | - | - | - |
| C0192 | + | - | - | - |
| C0193 | + | - | - | + |
| C0194 | + | - | - | - |
| C0195 | +++ | + | - | - |
| C0196^#^ | +++ | +++ | - | - |
| C0198 | +++ | - | - | - |
| C0201 | +++ | - | - | - |
| C0205 | + | - | - | - |
| C0206 | + | - | - | - |
| C0211 | + | - | - | - |
| C0215 | +++ | - | - | - |
| C0219^#^ | + | - | - | +++ |
| C0222 | + | - | - | - |
| C0223 | + | - | - | - |
| C0224 | + | - | - | - |
| C0225 | +++ | - | - | - |
| C0226 | + | - | - | - |
| C0228 | + | - | - | - |
| C0229 | + | - | - | - |
| C0230 | + | - | - | - |
| C0232 | + | - | - | - |
| C0233 | + | - | - | - |
| C0235 | + | - | - | + |
| C0236 | + | - | - | - |
| C0237 | + | - | - | - |
| C0239 | + | - | - | - |
| C0242 | + | - | - | - |
| C0243 | + | - | - | - |
| C0246 | + | - | - | - |
| C0247 | + | - | - | - |
| C0249 | + | - | - | - |
| C0250 | + | - | - | - |
| C0301 | + | - | - | - |
| C0302 | + | - | - | - |
| C0303 | + | - | - | - |
| C0304 | + | - | - | - |
| C0305 | + | - | - | - |
| C0306 | +++ | - | - | - |
| C0307 | + | - | - | - |
| C0311 | +++ | - | - | - |
| C0316 | + | - | - | - |
| C0320 | + | - | - | - |
| C0322 | + | - | - | - |
| C0323 | + | - | - | - |
| C0326 | + | - | - | - |
| C0327 | +++ | - | - | - |
| C0338 | + | - | - | - |
| C0339 | + | - | - | - |
| C0340 | +++ | - | - | - |
| C0341 | + | - | - | - |
| C0343 | + | - | - | - |
| C0345 | +++ | - | - | - |
| C0348 | +++ | - | - | - |
| C0349 | + | - | - | - |
| C0350 | + | - | - | - |
| C0351 | + | - | - | - |
| C0353 | + | - | - | - |
| C0355 | + | - | - | - |
| C0356 | + | - | - | - |
| C0358 | + | - | - | - |
| C0359 | + | - | - | - |
| C0360 | +++ | + | - | +++ |
| C0361 | +++ | - | - | - |
| C0363 | +++ | - | - | - |
| C0364 | +++ | - | - | - |
| C0366 | +++ | - | - | - |
| C0367 | +++ | + | - | - |
| C0368 | +++ | - | - | - |
| C0369^#^ | +++ | - | - | +++ |
| C0370^#^ | +++ | +++ | - | - |
| C0371 | +++ | - | - | - |
| C0372 | +++ | - | - | - |
| C0373 | +++ | - | - | - |
| C0375 | +++ | - | - | - |
| C0376 | +++ | - | - | - |
| C0377 | + | - | - | + |
| C0378 | +++ | - | - | - |
| C0379^#^ | +++ | +++ | - | - |
| C0380^#^ | + | + | - | - |
| C0381 | +++ | - | - | - |
| C0382 | +++ | - | - | - |
| C0383 | +++ | + | - | + |
| C0384 | +++ | + | - | - |
| C0385 | + | - | - | - |
| C0386 | +++ | - | - | - |
| C0388 | + | - | - | + |
| C0389^#^ | +++ | - | - | +++ |
| C0392 | +++ | - | - | - |
| C0393 | + | - | - | - |
| C0394 | +++ | - | - | - |
| C0397 | + | - | - | - |
| C0398 | +++ | - | - | + |
| C0399 | +++ | - | - | - |
| C0400 | +++ | - | - | - |
| C0401 | +++ | - | - | - |
| C0402 | +++ | - | - | - |
| C0403 | +++ | - | - | + |
| C0406 | + | - | - | - |
| C0407 | +++ | - | - | - |
| C0408 | +++ | + | - | - |
| C0409 | + | - | - | - |
| C0410 | +++ | - | - | - |
| C0411 | +++ | - | - | - |
| C0412 | +++ | - | - | - |
| C0413 | + | + | - | - |
| C0414 | + | - | - | - |
| C0415 | +++ | - | - | - |
| C0416 | +++ | - | - | - |
| C0417 | + | - | - | - |
| C0419 | +++ | - | - | + |
| C0420 | + | - | - | - |
| C0421 | +++ | + | - | + |
| C0422 | + | - | - | - |
| C0423 | +++ | - | - | - |
| C0424 | +++ | - | - | - |
| C0425 | +++ | - | - | + |
| C0426 | +++ | - | - | - |
| C0427 | + | - | - | - |
| C0428 | +++ | - | - | + |
| C0430 | +++ | - | - | - |
| C0431 | + | - | - | - |
| C0432 | + | - | - | - |
| C0433 | + | - | - | - |
| C0434 | +++ | - | - | - |
| C0438 | +++ | - | - | - |
| C0439 | +++ | - | - | - |
| C0440 | +++ | - | - | - |
| C0441 | +++ | - | - | - |
| C0442 | +++ | - | - | - |
| C0443 | + | - | - | - |
| C0444 | +++ | - | - | - |
| C0445 | +++ | - | - | - |
| C0447 | + | - | - | - |
| C0448 | +++ | - | - | - |
| C0449 | +++ | - | - | - |
| C0450 | +++ | - | - | - |
| C0454 | + | - | - | - |
| C0456 | +++ | - | - | - |
| C0458 | +++ | - | - | - |
| C0460 | +++ | - | - | - |
| C0461 | + | - | - | - |
| C0465 | + | - | - | - |
| C0466 | +++ | - | - | - |
| C0470 | +++ | - | - | - |
| C0471 | +++ | - | - | - |
| C0472 | +++ | - | - | + |
| C0474 | + | - | - | - |
| C0475 | + | - | - | - |
| C0476 | + | - | - | - |
| C0477 | +++ | - | - | - |
| C0478 | + | + | - | + |
| C0479 | + | - | - | - |
| C0480 | + | - | - | - |
| C0483 | + | - | - | - |
| C0486 | + | - | - | - |
| C0487 | +++ | - | - | - |
| C0488 | +++ | - | - | - |
| C0489 | +++ | - | - | - |
| C0490 | +++ | - | - | - |
| C0491 | + | - | - | + |
| C0492 | +++ | - | - | - |
| C0493 | +++ | - | - | - |
| C0494 | +++ | - | - | - |
| C0495 | +++ | - | - | - |
| C0497 | +++ | - | - | - |
| C0498 | +++ | - | - | + |
| C0499 | +++ | - | - | - |
| C0500 | +++ | - | - | - |
| C0501 | +++ | - | - | - |
| C0504 | +++ | - | - | - |
| C0505 | +++ | - | - | - |
| C0506 | + | - | - | - |
| C0510 | +++ | - | - | - |
| C0512 | + | - | - | - |
| C0513 | +++ | - | - | - |
| C0515 | +++ | - | - | - |
| C0516 | + | - | - | - |
| C0517 | +++ | - | - | - |
| C0519 | +++ | - | - | + |
| C0520 | + | - | - | + |
| C0523 | +++ | - | - | - |
| C0524 | +++ | - | - | - |
| C0525 | + | - | - | - |
| C0526 | +++ | - | - | - |
| C0527 | + | - | - | + |
| C0529 | +++ | - | - | - |
| C0530 | + | - | - | - |
| C0533 | + | - | - | - |
| C0535 | +++ | - | - | - |
| C0536 | + | - | - | + |
| C0539 | +++ | - | - | - |
| C0540 | + | - | - | - |
| C0544 | +++ | - | - | + |
| C0545 | + | - | - | - |
| C0546 | +++ | - | - | - |
| C0547 | +++ | - | - | - |
| C0548 | +++ | - | - | - |
| C0550 | + | - | - | + |
| C0551 | + | - | - | - |
| C0554 | + | - | - | - |
| C0555 | + | - | - | - |
| C0556 | + | - | - | - |
| C0557 | +++ | - | - | + |
| C0558 | + | - | - | - |
| C0559 | + | - | - | - |
| C0574 | +++ | - | - | - |
| C0577 | +++ | - | - | - |
| C0578 | +++ | - | - | - |
| C0579 | + | - | - | + |
| C0581 | + | - | - | - |
| C0584 | +++ | - | - | - |
| C0586 | +++ | - | - | + |
| C0589 | +++ | - | - | - |
| C0591 | +++ | + | - | + |
| C0592 | +++ | - | - | - |
| C0593 | +++ | - | - | + |
| C0594 | +++ | - | - | + |
| C0596 | + | - | - | - |
| C0597 | + | - | - | - |
| C0599 | +++ | + | - | - |
| C0602 | + | - | - | - |
| C0603 | +++ | - | - | - |
| C0604 | + | - | - | - |
| C0605 | + | - | - | - |
| C0606 | +++ | - | - | - |
| C0610 | +++ | - | - | - |
| C0613 | + | - | - | - |
| C0616 | + | - | - | - |
| C0619 | +++ | - | - | - |
| C0620 | + | - | - | - |
| C0623 | + | - | - | - |
| C0624 | +++ | - | - | - |
| C0626 | + | - | - | - |
| C0628 | +++ | - | - | - |
| C0632 | +++ | - | - | - |
| C0633 | +++ | - | - | - |
| C0635 | +++ | - | - | - |
| C0636^#^ | +++ | - | - | +++ |
| C0637 | + | - | - | - |
| C0638 | +++ | - | - | - |
| C0639 | +++ | - | - | - |
| C0641 | +++ | - | - | - |
| C0644 | +++ | - | - | - |
| C0650 | + | - | - | - |
| C0656 | +++ | - | - | - |
| C0660 | +++ | + | - | - |
| C0661^#^ | +++ | - | - | +++ |
| C0663 | + | + | - | - |
| C0664 | + | - | - | - |
| C0666 | +++ | - | - | - |
| C0667 | + | - | - | - |
| C0670 | + | - | - | - |
| C0671 | + | - | - | - |
| C0675 | + | - | - | - |
| C0676 | + | - | - | + |
| C0677^#^ | +++ | +++ | - | - |
| C0688 | + | - | - | - |
| C0689 | + | - | - | - |
| C0692 | +++ | - | - | - |
| C0699 | +++ | - | - | - |
| C0700 | + | - | - | - |
| C0701^#^ | +++ | + | - | - |
| C0702 | + | - | - | - |
| C0703 | + | - | - | - |
| C0704 | + | - | - | - |
| C0708 | + | - | - | - |
| C0711 | +++ | - | - | - |
| C0713 | + | - | - | - |
| C0716 | + | - | - | - |
| C0717 | +++ | - | - | - |
| C0719 | +++ | - | - | - |
| C0720 | + | - | - | - |
| C0721 | + | - | - | - |
| C0724^#^ | +++ | - | - | +++ |
| C0726 | + | - | - | - |
| C0731 | +++ | + | - | - |
| C0737 | + | + | - | - |
| C0739 | +++ | - | - | - |
| C0743 | + | - | - | - |
| C0744 | + | - | - | - |
| C0747 | +++ | - | - | - |
| C0748 | + | - | - | - |
| C0749 | + | - | - | - |
| C0751 | + | - | - | - |
| C0752 | + | - | - | - |
| C0754 | +++ | - | - | - |
| C0758 | + | - | - | - |
| C0759 | + | - | - | - |
| C0760 | + | - | - | - |
| C0764 | + | - | - | - |
| C0765 | +++ | - | - | - |
| C0766 | + | - | - | - |
| C0767 | + | - | - | - |
| C0769 | + | - | - | - |
| C0770 | + | - | - | - |
| C0771 | + | + | - | - |
| C0773 | + | - | - | - |
| C0777 | +++ | + | - | - |
| C0778 | + | - | - | - |
| C0779 | + | - | - | - |
| C0782 | + | - | - | - |
| C0783 | +++ | - | - | - |
| C0785 | +++ | - | - | - |
| C0786^#^ | +++ | - | + | +++ |
| C0787 | +++ | - | - | + |
| C0790 | + | - | - | - |
| C0792 | + | - | - | - |
| C0793 | + | - | - | - |
| C0795 | + | - | - | - |
| C0796 | + | - | - | - |
| C0797 | + | - | - | - |
| C0798 | + | - | - | - |
| C0802 | + | - | - | - |
| C0804 | +++ | - | - | - |
| C0805 | + | - | - | + |
| C0806 | + | - | - | - |
| C0807 | + | - | - | - |
| C0808 | + | - | - | - |
| C0809 | + | - | - | - |
| C0811 | +++ | - | - | + |
| C0813 | + | - | - | - |
| C0814 | +++ | + | - | - |
| C0816 | +++ | - | - | - |
| C0817 | +++ | - | - | - |
| C0819 | +++ | - | - | - |
| C0820 | + | + | - | - |
| C0822 | + | - | - | - |
| C0823 | + | - | - | - |
| C0824 | + | + | - | - |
| C0825 | + | - | - | + |
| C0827 | + | + | - | - |
| C0829 | + | - | - | - |
| C0831 | + | + | - | - |
| C0832 | + | - | - | - |
| C0833 | +++ | - | - | - |
| C0834 | + | - | - | - |
| C0835 | +++ | - | - | - |
| C0836 | + | + | - | - |
| C0837 | + | +++ | - | - |
| C0839 | + | - | - | - |
| C0840 | + | - | - | - |
| C0841 | +++ | - | - | - |
| C0844 | + | - | - | - |
| C0846 | +++ | - | - | - |
| C0850 | + | - | - | - |
| C0851 | + | - | - | - |
| C0858 | +++ | - | - | - |
| C0859 | + | - | - | - |
| C0860 | + | - | - | - |
| C0862 | + | + | - | - |
| C0865 | +++ | - | - | - |
| C0867 | +++ | - | - | - |
| C0869 | + | - | - | - |
| C0870 | + | - | - | + |
| C0873 | + | - | - | - |
| C0874 | + | - | - | - |
| C0880 | + | - | - | - |
| C0882 | + | + | - | - |
| C0883 | + | - | - | - |
| C0884 | + | - | - | - |
| C0886 | + | - | - | - |
| C0888 | + | - | - | - |
| C0890 | + | - | - | - |
| C0891 | + | - | - | + |
| C0892 | + | - | - | - |
| C0894 | +++ | - | - | +++ |
| C0895 | +++ | - | - | + |
| C0898 | + | - | - | - |
| C0901 | + | - | - | - |
| C0903 | +++ | + | - | - |
| C0907 | +++ | - | - | - |
| C0910 | + | - | - | - |
| C0912 | + | - | - | + |
| C0913 | +++ | - | - | - |
| C0914 | + | - | - | - |
| C0915 | +++ | - | - | - |
| C0917 | + | - | - | - |
| C0921^#^ | +++ | - | - | +++ |
| C0923 | +++ | - | - | - |
| C0924 | +++ | - | - | - |
| C0926 | +++ | - | - | - |
| C0927 | +++ | - | - | - |
| C0928 | + | - | - | - |
| C0929 | +++ | - | - | - |
| C0930 | + | - | - | - |
| C0931 | + | - | - | - |
| C0932 | +++ | - | - | - |
| C0933 | +++ | - | - | - |
| C0935 | + | - | - | - |
| C0937 | +++ | - | - | - |
| C0941 | + | - | - | - |
| C0942 | + | - | - | - |
| C0943 | + | + | - | - |
| C0944 | + | - | - | - |
| C0947 | + | - | - | - |
| C0949 | + | + | - | + |
| C0954 | + | + | - | - |
| C0958 | + | - | - | +++ |
| C0960 | + | - | - | - |
| C0963 | + | - | - | - |
| C0965 | +++ | + | - | + |
| C0966 | + | - | - | - |
| C0971 | + | - | - | - |
| C0973 | +++ | - | - | - |
| C0974 | + | - | - | + |
| C0976 | + | + | - | - |
| C0977 | + | - | - | - |
| C0981 | + | - | - | - |
| C0982 | +++ | - | - | - |
| C0985 | +++ | - | - | - |
| C0989 | +++ | - | - | - |
| C0990 | + | - | - | - |
| C0991 | +++ | - | - | - |
| C0992 | +++ | - | - | - |
| C0993 | + | - | - | + |
| C0995 | +++ | - | - | - |
| C0996 | +++ | - | - | - |
| C0997 | +++ | + | - | - |
| C1002 | + | - | - | - |
| C1006 | + | - | - | - |
| C1007 | + | - | - | - |
| C1012 | +++ | - | - | - |
| C1020 | + | - | - | - |
| C1022 | +++ | - | - | - |
| C1023 | + | - | - | - |
| C1025 | +++ | - | - | - |
| C1030 | +++ | - | - | - |
| C1031 | + | - | - | - |
| C1032 | + | - | - | - |
| C1037 | + | - | - | - |
| C1039 | + | - | - | - |
| C1040 | + | - | - | - |
| C1041 | + | - | - | - |
| C1042 | + | - | - | - |
| C1043 | + | + | - | - |
| C1044 | + | - | - | - |
| C1046 | + | - | - | - |
| C1047 | + | - | - | - |
| C1049 | + | - | - | - |
| C1050 | + | - | - | - |
| C1052 | + | + | - | - |
| C1054 | + | - | - | - |
| C1056 | + | - | - | - |
| C1057 | + | - | + | - |
| C1062 | + | - | - | - |
| C1065 | + | - | - | - |
| C1067 | + | - | - | + |
| C1068 | + | - | - | - |
| C1070 | + | - | - | - |
| C1072 | + | - | - | - |
| C1073 | + | + | - | - |
| C1075 | + | - | - | - |
| C1077 | + | + | - | - |
| C1082 | + | - | - | +++ |
| C1083 | + | - | - | - |
| C1085 | + | - | - | - |
| C1090 | + | - | - | - |
| C1097 | + | - | - | - |
| C1098 | + | - | - | - |
| C1099 | + | - | - | - |
| C1100 | + | - | - | - |
| C1104 | + | - | - | +++ |
| C1108 | + | - | - | - |
| C1110 | + | - | - | - |
| C1118 | + | - | - | - |
| C1119 | + | - | - | - |
| C1121 | + | - | - | - |
| C1123 | + | - | - | - |
| C1124 | + | - | - | - |
| C1126 | + | - | - | - |
| C1135 | + | - | - | - |
| C1136 | + | - | + | - |

Supplementary Table 2. The association of camel age, gender, birth place, migration status, pregnancy status (A) and breed species (B) with MERS-CoV or MERS/HKU8r-CoV co-infection cases distribution. Only samples that are MERS-CoV RBD ELISA positive were calculated. The association p value was calculated using Chi square test followed with Yates correlation two-tailed test and Fisher’s exact test.

| **A** |  |  |  |  |  |
| --- | --- | --- | --- | --- | --- |
| County | Breed | MERS NP Positive | | HKU8 NP Positive | |
|  |  | n/m | % (95% CI) | n/m | % (95% CI) |
| Laikipia | Pakistan /Mixture | 0/14 | 0 | 0/14 | 0 |
|  |  |  |  |  |  |
| Turkana | Turkana | 0/74 | 0 | 15/74 | 20.3 (13.4-31.5) |
| West pokot |  |  |  |  |  |
| Baringo |  |  |  |  |  |
| Samburu | Gabbra /Rendille | 12/228 | 5.3 (3.4-9.4) | 26/228 | 11.4 (8.2-16.5) |
| Isiolo |  |  |  |  |  |
| Marsabit |  |  |  |  |  |
| Wajir | Somali | 27/260 | 10.4 (7.5-15) | 25/260 | 9.6 (6.9-14.1) |
| Tana River |  |  |  |  |  |
| Mandera |  |  |  |  |  |
| Makueni |  |  |  |  |  |
| Kitui |  |  |  |  |  |
| Garissa |  |  |  |  |  |
|  |  | p=0.003 | | p=0.039 | |
|  |  |  |  |  |  |
| **B** |  |  |  |  |  |
| Factors | | MERS NP+% | p value | HKU8 NP+% | p value |
| Age | Immature | 4.90% | 0.524 | 7.80% | 0.26 |
|  | Mature | 7.20% |  | 12.30% |  |
| Gender | Male | 6.50% | 1 | 10.90% | 0.988 |
|  | Female | 6.80% |  | 11.60% |  |
| Birth place | Not home | 6.90% | 1 | 13.80% | 0.284 |
|  | Home | 6.70% |  | 10.30% |  |
| Migration | No | 5.90% | 0.377 | 12.90% | 0.614 |
|  | Yes | 8.50% |  | 10.90% |  |
| Pregnancy | No | 5.50% | 0.191 | 13.40% | 0.168 |
|  | Yes | 9.20% |  | 8.60% |  |
